# Kaposi's Sarcoma-Associated Herpesvirus ORF57 Protein Binds and Protects a Nuclear Noncoding RNA from Cellular RNA Decay Pathways

**DOI:** 10.1371/journal.ppat.1000799

**Published:** 2010-03-05

**Authors:** Brooke B. Sahin, Denish Patel, Nicholas K. Conrad

**Affiliations:** Department of Microbiology, University of Texas Southwestern Medical Center, Dallas, Texas, United States of America; University of Southern California School of Medicine, United States of America

## Abstract

The control of RNA stability is a key determinant in cellular gene expression. The stability of any transcript is modulated through the activity of cis- or trans-acting regulatory factors as well as cellular quality control systems that ensure the integrity of a transcript. As a result, invading viral pathogens must be able to subvert cellular RNA decay pathways capable of destroying viral transcripts. Here we report that the Kaposi's sarcoma-associated herpesvirus (KSHV) ORF57 protein binds to a unique KSHV polyadenylated nuclear RNA, called PAN RNA, and protects it from degradation by cellular factors. ORF57 increases PAN RNA levels and its effects are greatest on unstable alleles of PAN RNA. Kinetic analysis of transcription pulse assays shows that ORF57 protects PAN RNA from a rapid cellular RNA decay process, but ORF57 has little effect on transcription or PAN RNA localization based on chromatin immunoprecipitation and in situ hybridization experiments, respectively. Using a UV cross-linking technique, we further demonstrate that ORF57 binds PAN RNA directly in living cells and we show that binding correlates with function. In addition, we define an ORF57-responsive element (ORE) that is necessary for ORF57 binding to PAN RNA and sufficient to confer ORF57-response to a heterologous intronless β-globin mRNA, but not its spliced counterparts. We conclude that ORF57 binds to viral transcripts in the nucleus and protects them from a cellular RNA decay pathway. We propose that KSHV ORF57 protein functions to enhance the nuclear stability of intronless viral transcripts by protecting them from a cellular RNA quality control pathway.

## Author Summary

In order to replicate efficiently, a virus must ensure that its genes are properly expressed in the context of an infected host cell. Recent work has demonstrated that eukaryotic cells have RNA quality control pathways that degrade improperly processed, aberrant RNAs. Our published findings using an unusual Kaposi's sarcoma-associated herpesvirus (KSHV) nuclear RNA, called PAN RNA, have suggested that intronless polyadenylated transcripts are subject to such a quality control system. Because most KSHV genes lack introns, we hypothesized that KSHV must have evolved mechanisms that bypass this quality control system. In support of this idea, we show that the ORF57 protein, a multifunctional enhancer of KSHV gene expression, binds to and stabilizes PAN RNA. We further define an element called the ORF57-responsive element (ORE) in PAN RNA that is necessary for ORF57-binding and activity on PAN RNA. In addition, we show that the ORE is sufficient to confer ORF57-responsiveness to a heterologous intronless mRNA, but not its spliced counterpart. These observations substantiate the model that ORF57 enhances KSHV gene expression by protecting viral transcripts from host RNA surveillance pathways. More broadly, these data suggest that viruses producing intronless nuclear RNAs require mechanisms to evade host quality control mechanisms.

## Introduction

Post-transcriptional events in mRNA biogenesis are of central importance to the fidelity and regulation of gene expression. Cellular factors regulate nearly every step of RNA metabolism including transcription elongation, RNA splicing, 3′ end formation, nuclear export, translation, etc. In fact, genome-wide profiling experiments demonstrate that a significant percent of the observed changes in RNA levels are dictated by regulation of the stability of a transcript rather than its transcription (e.g. [1],[2]). RNA half-life can be modulated directly, through the activities of regulatory stabilizing or destabilizing protein factors or small RNAs [3]–[5]. In addition, RNA quality control pathways ensure aberrant transcripts are less stable than their functional counterparts [6]. Given the importance of these pathways for gene expression, it is no surprise that viruses have evolved mechanisms to counteract pathways that otherwise would lead to the destruction of viral transcripts [3],[7].

The Kaposi's sarcoma-associated herpesvirus (KSHV) is a member of the gammaherpesvirus family that causes Kaposi's sarcoma, a common AIDS-associated malignancy, as well as the lymphoproliferative disorders primary effusion lymphoma (PEL) and some cases of multicentric Castleman's disease (MCD) [8]–[10]. The life cycle of KSHV includes a latent phase in which the viral DNA is maintained in infected host cells as a circular episome. During latency, few viral genes are expressed and no viral replication occurs. When the KSHV lytic phase is reactivated, a well-regulated cascade of gene expression is initiated by the viral transactivator ORF50 (Rta) resulting in infectious virus production [11]–[13]. Like all herpesviruses, the KSHV genome is nuclear and its genes are expressed utilizing the host cell transcription, RNA processing, and translation machinery. In many respects, KSHV genes resemble those of their host; that is, they have canonical promoter elements, 3′-end formation signals, and consensus pre-mRNA splice sites.

However, KSHV genes differ from canonical cellular genes in several relevant ways. Some transcripts are bicistronic, KSHV introns are smaller than the average size of a mammalian intron, and genes are more closely arranged in the genome than host genes. Most importantly for the present work, ∼70% of KSHV genes lack introns [14], whereas most human protein-coding genes contain multiple introns [15]. This difference in gene structure has implications for the expression of viral genes. The presence of an intron in a pre-mRNA and/or the changes in ribonucleoprotein particle (RNP) composition that result from splicing promote the efficiency of almost every stage of gene expression, including transcription initiation and elongation, 3′-end formation, mRNA export, RNA localization, and translation [16]–[27]. As a result, transgenes containing an intron are often expressed at significantly higher levels than those same genes lacking an intron [18]. To compensate for the lack of introns or splicing, viruses that express unspliced or intronless transcripts have evolved mechanisms that promote efficient gene expression in the absence of splicing [28]–[33].

KSHV encodes a viral post-transcriptional regulator of gene expression called ORF57 (Mta, KS-SM) that is essential for viral replication [34],[35]. ORF57 is a member of a conserved family of herpesvirus proteins that post-transcriptionally enhance gene expression [29]–[33]. ORF57 has been implicated in a variety of steps of RNA biogenesis from transcription to translation and it increases the efficiency of intronless gene expression [30],[31],[36],[37]. ORF57 has been reported to interact with ORF50 and to enhance transcription in a promoter and cell-type specific manner [37]–[39]. In addition, ORF57 binds cellular export factors and promotes the nuclear export of at least a subset of intronless viral mRNAs [40]–[43]. Unlike the herpes simplex homolog (HSV) ICP27, which contributes to host gene shut-off by inhibiting splicing, ORF57 promotes the splicing of some viral mRNAs [35],[44], and splicing activity is also seen with the Epstein-Barr virus (EBV) ORF57 homolog, SM [45]. ORF57 has further been suggested to play a role in translation of an internal ribosome entry site-containing reporter [46]. Thus, ORF57 is a multifunctional regulator of mRNA biogenesis that may, in part, compensate for the lack of introns in viral gene expression.

ORF57 is critical for the accumulation of the polyadenylated nuclear (PAN) RNA (nut1, T1.1) [34],[35],[37],[42], a non-coding nuclear transcript that accumulates to high levels during the lytic phase of viral infection [47],[48]. The PAN RNA promoter is ORF50-dependent [49],[50], and PAN RNA accumulation further depends on the activity of a 79-nucleotide (nt) RNA element, called the ENE [51]–[53]. Mechanistically, the ENE interacts in cis with the poly(A) tail of PAN RNA resulting in the sequestration of the poly(A) tail from exonucleases. Detailed kinetic analysis of the effects of the ENE on PAN RNA decay in transfected cells showed that PAN RNA is subject to two kinetically distinguishable decay pathways, one with a very short half-life (10–20 min) and another with a longer half-life (3–5 hrs). ENE-lacking or ENE-mutant PAN transcripts are more likely to be degraded in the rapid RNA decay pathway than are their wild-type ENE containing counterparts. Because the ENE is sufficient to increase the nuclear accumulation of heterologous intronless transcripts, we further proposed that this rapid decay pathway is part of a nuclear RNA surveillance system that rapidly degrades inefficiently exported mRNAs.

ORF57-mediated enhancement of the exclusively nuclear PAN RNA suggests that it may be involved in inhibiting the proposed RNA surveillance mechanism. Here, we test this idea and find that ORF57 stabilizes PAN RNA, particularly those transcripts that lack the ENE. We see no ORF57-dependent effect on RNA polymerase II (pol II) density on the PAN RNA gene nor does ORF57 lead to PAN RNA export. Therefore, we conclude that the observed stability enhancement constitutes the major effect of ORF57 on PAN RNA accumulation. In addition, ORF57 binds PAN RNA directly in vivo and a deletion of the 5′ portion of PAN RNA, dubbed the ORF57-responsive element (ORE), reduces ORF57 binding and ORF57 response. We show that tethering of ORF57 to an ORE-deleted PAN RNA restores ORF57-mediated up-regulation. Finally, we show that the ORE is sufficient to confer increased ORF57-response to a heterologous intronless β-globin mRNA, but not its spliced counterpart. We conclude that ORF57 protects viral transcripts from the same cellular RNA decay pathway that the ENE protects from in cis and that its stabilization activity is dependent on ORF57 binding to target RNAs.

## Results

### ORF57 protects transcripts from rapid decay

**Figure 1:**
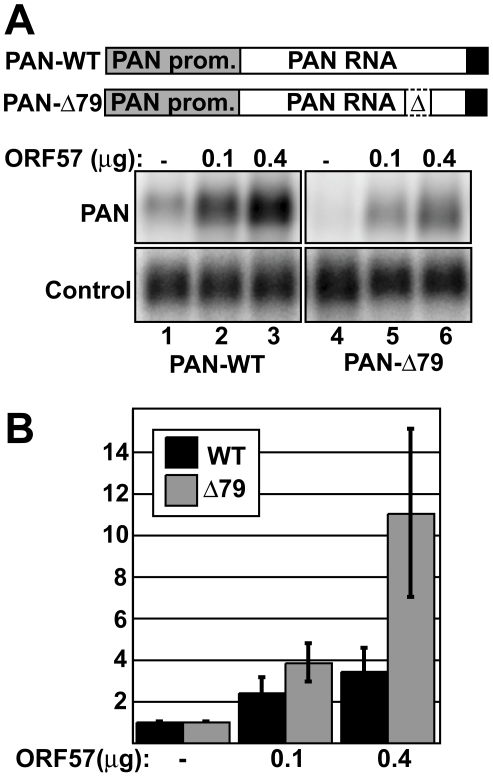
ORF57 preferentially enhances the levels of an unstable nuclear RNA. (A) *Top*, schematic diagram of the constructs used. Both are driven by the PAN promoter (gray) and have the PAN 3′-end formation signals (black). PAN-Δ79 has the 79-nt ENE sequence deleted. *Bottom*, representative northern blot showing a dose-dependent response of PAN-WT and PAN-Δ79 to ORF57. Cells were co-transfected with PAN and ORF57 expression plasmids as indicated. Because these constructs use the ORF50-dependent PAN promoter, an ORF50 expression plasmid was also co-transfected. Control panels show signal from a co-transfected plasmid that controls for transfection and loading efficiencies. (B) Quantitation of dose-dependent experiments shown in (A). Values are normalized to the no ORF57 control lanes; error bars show standard deviation (*n = 4*).

If ORF57 protects transcripts from RNA decay pathways in vivo, we reasoned that the effects of ORF57 would be more pronounced on unstable ENE-lacking transcripts than on their ENE-containing counterparts. To test this idea, we compared the RNA levels of PAN RNA containing the ENE (PAN-WT) to PAN RNA lacking the ENE (PAN-Δ79) in the absence of ORF57 or in its presence. We transfected HEK293 cells with constructs that express PAN-WT or PAN-Δ79 and co-transfected ORF57-expression constructs at two concentrations or empty vector. After ∼18-24 hours, we extracted total RNA, and quantified relative RNA levels by northern blot (Figure 1). Consistent with published results [34],[35],[37],[42], ORF57 increases wild-type PAN RNA levels in a dose-dependent fashion (Figure 1A, lanes 1–3). Quantitation of these data show that, at the highest ORF57 concentration tested, PAN RNA is ∼3.4-fold more abundant (Figure 1B). As predicted from our model, ENE-lacking transcripts show an even greater response to ORF57, ∼11-fold (lanes 4–6 and Figure 1B). Because the ENE is involved in RNA stability, these results are consistent with the conclusion that ORF57 increases the half-life of PAN RNA.

**Figure 2:**
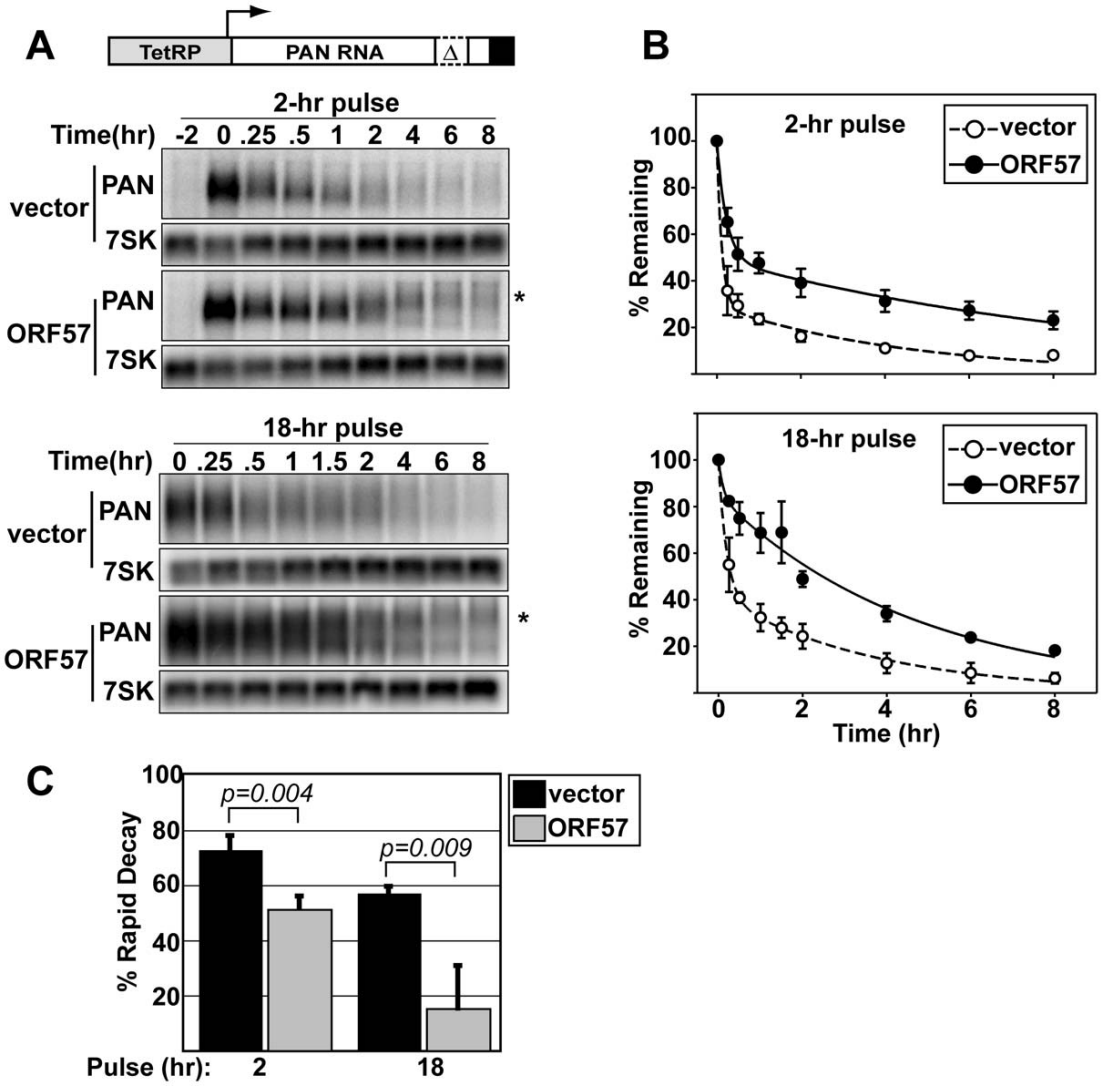
ORF57 protects transcripts from rapid RNA decay. (A) *Top*, schematic diagram of TRP-Δ79 construct, which contains the Tet-responsive promoter driving PAN-Δ79. *Bottom*, representative northern blots showing typical transcription pulse data. The upper panels show data from a two-hour pulse in the presence or absence of ORF57 as indicated. The “-2” lanes are samples taken prior to the pulse. The lower panels show the results from an 18-hr transcription pulse. The asterisks mark the mobility of hyperadenylated transcripts. Cellular 7SK serves as a loading control. (B) Regression analysis of the transcription pulse data. Curves are two-component exponential decay curves as previously described [51]; error bars are standard deviation (*n = 3*). (C) The percent of transcripts undergoing rapid decay was derived using regression analyses. Other kinetic parameters are given in Table S1 and Figure S1.

To directly examine the effects of ORF57 on PAN RNA half-life, we employed a transcription pulse strategy [54]. In these experiments, we transfected HEK293 Tet-off advanced (293TOA) cells with TRP-Δ79, a plasmid that expresses the ENE-lacking PAN-Δ79 transcript from a tetracycline-responsive promoter [52]. In 293TOA cells, transcription from this promoter is turned off in the presence of doxycycline (dox, a tetracycline analog), and is induced in its absence. In our initial experiments, we examined the effects of ORF57 on PAN RNA decay after a two-hour transcription pulse (Figure 2). As expected, TRP-Δ79 RNA was undetectable prior to transcription pulse, but can be detected after two hours in dox-free media (Figure 2A, top panels). Examination of the decay profiles clearly shows an increase in RNA stability when ORF57 is expressed (Figure 2B, top). Interestingly, the mobility of a portion of remaining transcripts after transcription shut-off is reduced while others show increased mobility. We have determined that these mobility changes are due to differences in poly(A) tail length (data not shown). The relationship between changes in poly(A) length and ORF57 function is currently under investigation and will be described elsewhere (see Discussion). We also examined the effects of ORF57 on TRP-WT, a PAN expression construct containing the ENE [52]. However, in 293TOA cells this plasmid produced an extremely stable transcript (t_1/2_>24hr), impractical for use in decay assays. Overall, these data demonstrate that ORF57 increases the half-life of unstable PAN RNA transcripts.

Previous studies showed that PAN RNA is subject to two decay pathways with different kinetic properties [51],[52]. That is, one pool of PAN RNA transcripts is degraded very rapidly with half-lives of ∼10-20 minutes, while another pool of transcripts is degraded more slowly (t_1/2_ ∼3-5hrs). The presence of the ENE appears to protect transcripts from the rapid decay system resulting in a decrease in the fraction of transcripts that are observed in this population. Consistent with these published findings, the decay profiles in Figure 2B are nicely fit by two-component exponential decay curves where the two components represent the two pools of transcripts. Using regression analysis, we can determine the decay parameters in PAN RNA degradation, including the fraction of transcripts undergoing rapid decay and the half-life of each population. Because ENE-lacking transcripts are preferentially up-regulated by ORF57, we predicted that, like the ENE, ORF57 expression would decrease the fraction of PAN transcripts in the rapid RNA decay pathway.

To test this idea, we performed regression analysis of the data for TRP-Δ79 RNA decay in the presence or absence of ORF57 (Figure 2B, Table S1 and Figure S1). Examination of the kinetic parameters shows that in the absence of ORF57, 73% of the transcripts are in the rapidly degrading population (t_1/2_ ∼7.8 min) (Figure 2C). In contrast, only 51% of the transcripts are degraded rapidly when ORF57 is co-expressed, a statistically significant decrease. Because the more slowly degrading transcripts accumulate over time, the fraction of transcripts observed in the rapidly degrading pool decreases when longer transcription pulse times are employed [51]. If ORF57 decreases the fraction of transcripts that degrade rapidly, it follows that the observed rapidly degrading fraction would decrease more quickly when ORF57 is present. Indeed, the effects of ORF57 on TRP-Δ79 RNA decay are even more apparent after an 18-hour transcription pulse (Figure 2A and Figure 2B). In this case, the apparent half-life (i.e. the time difference at 50% remaining, Figure 2B) is increased ∼8-fold. More importantly, the percent of transcripts degrading rapidly in the presence of ORF57 is reduced to 15%, while 57% is rapidly degraded in its absence (Figure 2C). Taken together, these data strongly argue that ORF57 enhances PAN RNA levels by protecting it from a rapid cellular RNA decay pathway.

### ORF57 up-regulation of PAN RNA occurs after transcription initiation

**Figure 3:**
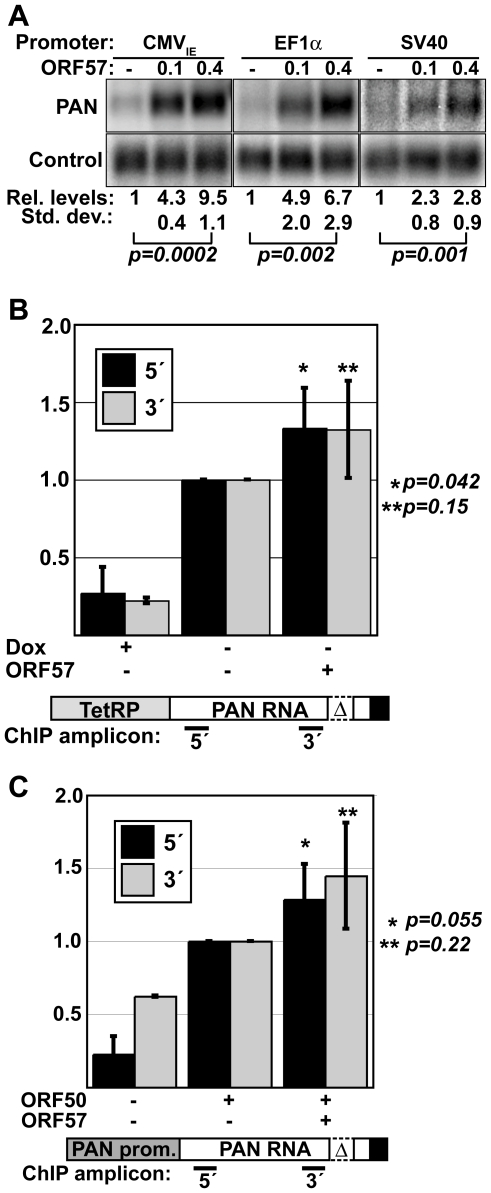
PAN RNA is posttranscriptionally up-regulated by ORF57. (A) Northern blots showing a dose-dependent response of PAN RNA driven by the CMV_IE_, EF1α, and SV40 promoters. Relative values and standard deviations are shown below; p values are given for a comparison of the 0 and 0.4 µg ORF57 quantities (CMV_IE_, *n = 3*; EF1a and SV40, *n = 5*). (B) Pol II ChIP results using TRP-Δ79. ChIP assays were done in the presence of dox or in its absence in cells transfected with or without ORF57 as indicated. PCR amplicons were nt 93-147 (5′) and 777-863 (3′) relative to the transcription start site. The diagram shown below is not to scale. Quantitation and background correction are described in the Materials and Methods section (PAN 5′: +dox, *n = 4*, +/−ORF57, *n = 5*; PAN 3′: +dox *n = 2*, +/− ORF57 *n = 3*). P-values are shown for the +/−ORF57 data sets. (C) Pol II ChIP results with PAN-Δ79; details are the same as (B). (PAN 5′: *n = 4*; PAN 3′: *n = 2*). The increased signal for the 3′ samples in the –ORF50 control is a result of lower signal for the 3′ amplicon in the +ORF50 samples and is not due to an increase in the amount of background signal (data not shown).

Previous reports suggested that ORF57 enhances transcription rates of specific promoters in certain cell types, including the PAN RNA promoter in 293 cells [37]–[39]. Even though we see an increase in PAN RNA half-life in the presence of ORF57, it remains possible that a significant portion of the up-regulation of PAN RNA by ORF57 is at the level of RNA synthesis rather than decay. To test the effects of ORF57 on transcription initiation, we initially examined the response of PAN RNA to ORF57 from three different promoters (Figure 3A). Consistent with the results of others, PAN RNA expression from the cytomegalovirus immediate early (CMV_IE_) promoter is responsive to ORF57 [36],[37],[39]. We extended this analysis by examining PAN RNA steady-state levels driven by the cellular elongation factor 1α (EF1α) and viral SV40 promoters (Figure 3A). Each of these constructs is 3′ processed using the PAN RNA cleavage and polyadenylation signals. For every promoter tested, ORF57 increased PAN RNA levels in a dose-dependent fashion supporting a post-transcriptional role for ORF57. Interestingly, the magnitude of the change differs among the constructs and this does not necessarily correlate with the strength of each promoter. For example, as judged by overall RNA levels, the PAN and SV40 promoters are the strongest and weakest promoters, respectively (Figure S2). However, PAN-WT RNA driven by each of these promoters is increased by similar margins at the highest ORF57 expression levels (3.5-fold and 2.8-fold) (Figure 1B and Figure 3A). In contrast, the CMV_IE_ and EF1α promoters both show greater increases in steady-state levels in response to ORF57 (9.5 and 6.7-fold, respectively). These data suggest that the effect of ORF57 on PAN RNA is not due to any promoter-specific element, but that magnitude of the ORF57 enhancement may be linked to a qualitative difference in these promoters.

To further test the effects of ORF57 on PAN RNA transcription, we examined the pol II density on the PAN RNA gene by chromatin immunoprecipitation assays (ChIP). In these experiments, ChIP was performed using antibodies to RNA polymerase II and the relative levels of co-immunoprecipitating DNA were compared. We examined DNA from the 5′ and 3′ ends of PAN RNA (Figure 3B) to assess polymerase density across the PAN gene. First, we determined the relative polymerase density on TRP-Δ79 in the presence and absence of ORF57 (Figure 3B) and saw only a slight increase in polymerase density on either the 5′ or 3′ portion of the PAN gene when ORF57 was co-expressed (∼1.3-fold). As expected, in the presence of dox, the polymerase density significantly decreases. Second, we assayed polymerase density on the PAN RNA gene when transcription is driven from the PAN promoter (Figure 3C). Because this promoter depends on the ORF50 viral transactivator, we included samples lacking ORF50 as negative controls. Comparing samples containing or lacking ORF57, we observed little difference in polymerase density at the 5′ end of the PAN gene. Similarly, we saw minimal ORF57-dependent difference in signal from the 3′ end of PAN gene, although there is lower signal in this sample (data not shown). Taken together, these data strongly support the conclusion that the predominant effect of ORF57 on PAN RNA steady-state levels occurs subsequent to transcription initiation.

### ORF57 does not induce the cytoplasmic accumulation of PAN RNA

**Figure 4:**
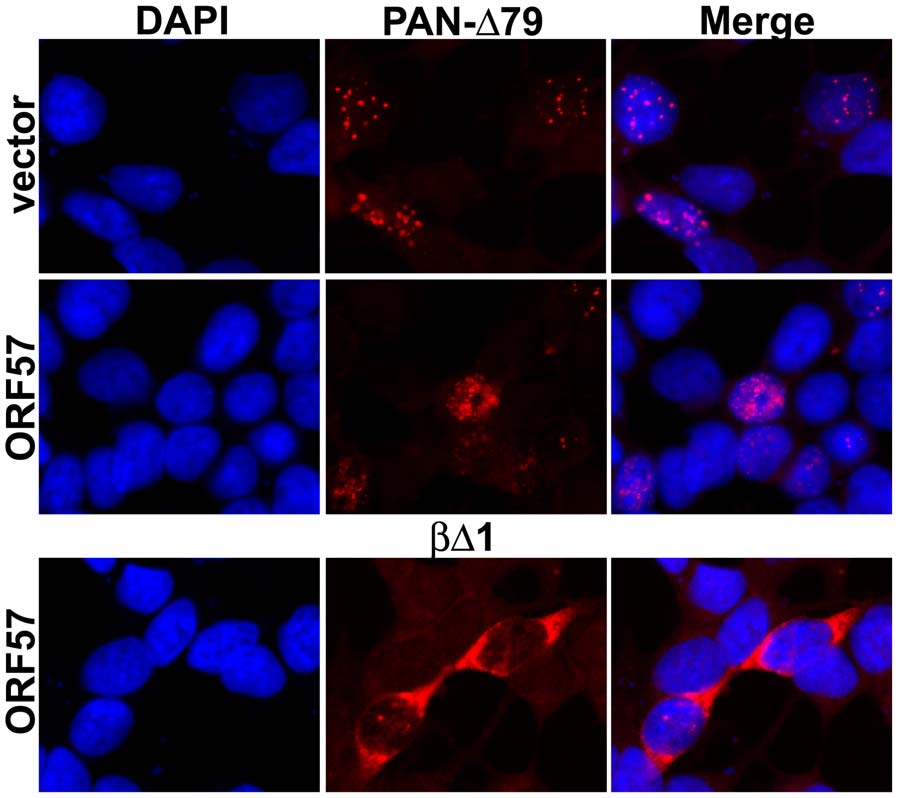
PAN-Δ79 RNA remains nuclear in the presence of ORF57. *Top*, in situ hybridization to PAN-Δ79 in transiently transfected HEK293 cells (middle panels) shows that PAN RNA remains nuclear in the presence or absence (vector) of ORF57 as indicated. Nuclei are stained with DAPI (left) and merged images are shown (right). *Bottom*, *c*ytoplasmic signal from a co-transfected β-globin reporter construct (βΔ1; Figure 8) serves as a control for maintenance of cytoplasmic RNA. The βΔ1 localization was unaffected by ORF57 (data not shown).

ORF57, like its homologs in other herpesviruses, has been implicated in the nuclear export of intron-lacking viral mRNAs [29], [32], [40]–[42],[55]. Because the machinery involved in RNA decay differs between the cytoplasm and the nucleus, it stands to reason that a change in subcellular localization would affect RNA decay profiles. Therefore, we performed in situ hybridization to verify that PAN RNA remains nuclear in the presence of ORF57. As shown in Figure 4, PAN-Δ79 RNA localizes to faint spots in the nucleus (top panels), with a few cells demonstrating a more diffuse pattern (data not shown and Figure S3). In the presence of ORF57, PAN-Δ79 RNA is also observed strictly in the nucleus (Figure 4, middle panels). The signal intensity, the number of cells showing signal above background, and the percentage of cells with diffuse nuclear staining were all increased in the presence of ORF57 and account for the higher levels of PAN-Δ79 in the presence of ORF57 (data not shown). Importantly, no cytoplasmic PAN RNA signal was detected. In addition, TRP-Δ79 RNA was observed exclusively in the nucleus (Figure S3), as was wild-type PAN RNA driven from either the PAN or TRP promoters (data not shown). As an additional control, since many in situ hybridization protocols will “wash away” cytoplasmic RNA, we tested whether we could detect cytoplasmic signal by examining the localization of a spliced β-globin reporter mRNA. In this case, cytoplasmic signal was observed (Figure 4, bottom panels). These results demonstrate that the increase in PAN RNA stability in the presence of ORF57 is not the result of PAN RNA export to the cytoplasm. Because ORF57 enhances RNA stability, but has little or no effect on PAN RNA export or transcription, we conclude that the effect of ORF57 on PAN RNA accumulation is the result of increased nuclear RNA stability.

### ORF57 binds PAN RNA directly in living cells

ORF57 could stabilize PAN RNA by one of two non-mutually exclusive mechanisms. First, ORF57 may inhibit the activity of RNA decay enzymes, either by binding and inactivating them directly or by decreasing their expression. Second, ORF57 may interact with transcripts and protect the bound RNAs, directly or indirectly, from decay enzymes. The second model predicts that ORF57 is in a complex with PAN RNA and that this binding is necessary for protection by ORF57. Even though a previous report suggested that ORF57 did not bind to PAN RNA in vitro [56], we investigated whether ORF57 bound to PAN RNA in vivo.

**Figure 5:**
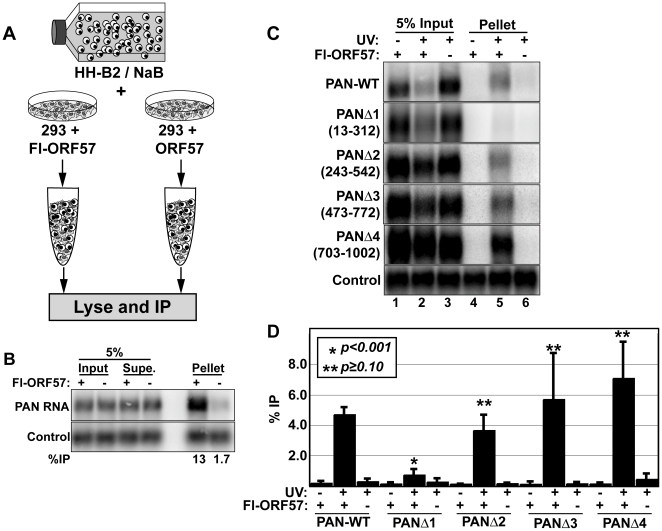
ORF57 binds PAN RNA in transfected cells. (A) Cell mixing experiment; details described in the text. (B) Northern blot of input (5%), supernatant (5%), and pellet (100%) RNA from a cell-mixing experiment. The control is an exogenously added transcript that controls for RNA recovery after immunoprecipitation. (C) Northern blots for PAN RNA showing 5% of the input RNA and 100% of the pellets from a UV cross-linking experiment. The lanes marked Fl-ORF57 “–”were transfected with an ORF57 expression plasmid lacking a Flag-tag. The nucleotides deleted in each of the PAN RNA variants are listed in parentheses to the left of each panel. One representative control is shown; the control is the same as in (B). (D) Quantitation of the results from UV cross-linking experiments. Average % immunoprecipitation is shown with error bars indicating standard deviation (PAN-WT, *n = 4*; PANΔ1, PANΔ3, PANΔ4, *n = 3*; PANΔ2, *n = 2*). The p-values compare the percent immunoprecipitation of the +UV/+Fl-ORF57 to that of PAN-WT.

One difficulty in examining RNA-protein interactions using co-immunoprecipitation techniques is that RNA-protein complexes frequently reassort in cellular extract [57],[58]. That is, an RNA-binding protein will associate with specific transcripts in an extract that were not bound in vivo. Therefore, in order to conclude that an RNA-protein interaction occurs in cells, it is imperative to test whether a given RNA-protein complex forms subsequent to lysis. To do this, we employed a “cell-mixing” experiment [57],[58] (Figure 5A). We induced lytic reactivation of KSHV in HH-B2 cells, a latently infected PEL cell line, with sodium butyrate (NaB) to initiate the expression of lytic genes including PAN RNA. After 24 hours, we mixed the lytically reactivated cells with HEK293 cells transfected with Flag-tagged ORF57 (Fl-ORF57) or with untagged ORF57 expression constructs. After combining intact cells, the cells were lysed and subjected to immunoprecipitation (IP) with anti-Flag antibodies. PAN RNA, which is exclusively derived from the HH-B2 cells, was efficiently and specifically immunoprecipitated with the anti-Flag beads (Figure 5B). Because PAN RNA is not produced in the same cells as the Fl-ORF57, this result clearly indicates that ORF57 interacts with RNAs in cell lysate and that RNAs that co-immunoprecipitate with ORF57 do not necessarily reflect RNP composition in vivo. As a result, caution must be taken in the interpretation and experimental design of RNA immunoprecipitation experiments with ORF57.

To control for ORF57-RNA reassortment in cell extract, we employed an ultraviolet light (UV) cross-linking protocol [57]. We transfected HEK293 cells with PAN-WT and Fl-ORF57 or untagged ORF57 expression plasmids and exposed the cells to UV light to covalently cross-link protein to RNA. Cells were lysed under stringent conditions and ORF57 was immunoprecipitated with anti-Flag antibodies. Subsequently, we detected PAN RNA signal by northern blot and quantitated this signal as a percent of input RNA (Figure 5C and 5D). Using this procedure, PAN RNA is immunoprecipitated from extracts containing Fl-ORF57, but not the untagged control (compare lanes 5 and 6, top panel). Most importantly, because living cells were exposed to UV, a UV-dependent interaction reflects an interaction in cells. Because the “no UV” control shows little detectable signal (lane 4), we conclude that ORF57 associates with PAN RNA in cells. Moreover, UV cross-linking is limited to interactions in which the RNA and protein are in close contact, so we can further conclude that ORF57 binds directly to PAN RNA in vivo.

Little is known about the requirements for ORF57 recruitment or RNA-binding in vivo, so we tested whether cis-acting elements in PAN RNA are necessary for the ORF57-PAN RNA interactions. We utilized a series of four previously described PAN RNA expression constructs with 300-nt overlapping deletions in PAN RNA, called PANΔ1-Δ4 [53]. ORF57 cross-linked to PANΔ2, PANΔ3, and PANΔ4 with similar efficiency as PAN-WT (Figures 5C and 5D). In contrast, cross-linking to PANΔ1 RNA was only slightly higher than background signal. We previously reported that PANΔ1 RNA is expressed at lower levels than PAN-WT RNA [53] (see below). To verify that the lower expression of PANΔ1 was not responsible for its lack of ORF57 binding, we performed experiments in which we transfected less of the PAN-WT expression plasmid, to render its steady-state levels similar levels to PANΔ1 (data not shown). In this case, efficient binding to PAN-WT was maintained, so we conclude that reduced ORF57-PANΔ1 cross-linking is not due to lower PANΔ1 expression levels. These results demonstrate that sequences near the 5′ end of PAN RNA are necessary for efficient cross-linking of ORF57 to PAN RNA in cultured cells.

### ORF57 binding correlates with ORF57 activity

**Figure 6:**
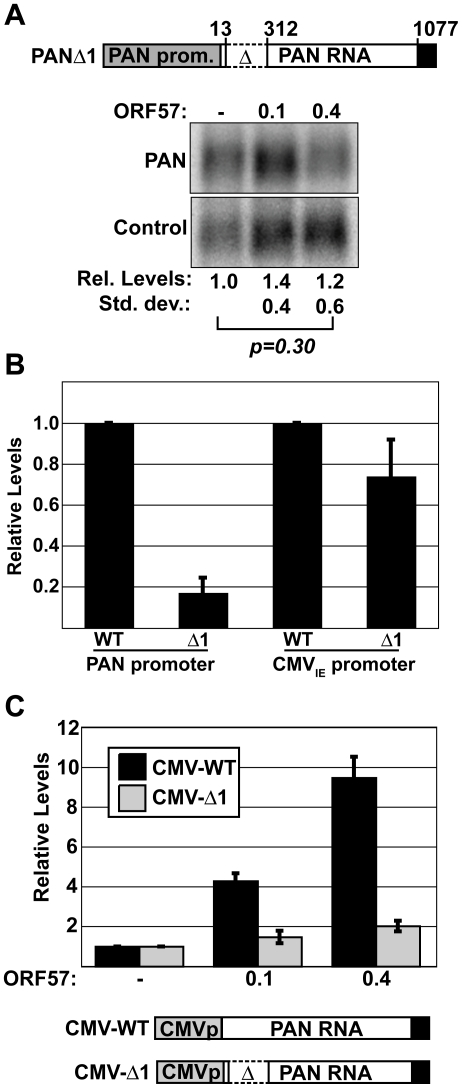
PANΔ1 is unresponsive to ORF57. (A) *Top*, diagram of PANΔ1 construct. *Below*, northern blots showing the lack of a dose-dependent response of PANΔ1 RNA to ORF57. Relative values and standard deviations are shown below (*n = 8*). (B) Comparison of the expression levels of PANΔ1 relative to PAN-WT driven from either the PAN promoter (left, *n = 3*) or the CMV_IE_ promoter (right, *n = 4*). (C) Quantitation of the relative ORF57-responsiveness of CMV-WT and CMV-Δ1, which are diagrammed below (*n = 3*). Data displayed in both (B) and (C) are from quantitative northern blot experiments.

The model that ORF57-RNA interactions are required for activity predicts that diminished binding of ORF57 to PANΔ1 will result in decreased ORF57 activity. Therefore, we examined the accumulation of PANΔ1 in the presence of increasing amounts of ORF57 (Figure 6A). Under the same conditions in which ORF57 increases PAN-WT by 3.4-fold and PAN-Δ79 by 11-fold (Figure 1), we observe no statistically significant change in PANΔ1 levels in the presence of ORF57. Thus, the loss of ORF57 binding to PANΔ1 correlates with loss of PAN RNA up-regulation. Taken with data presented below, our results show that the 5′ end of PAN RNA (nt 1–312) contains an ORF57-responsive element (ORE).

The loss of binding of ORF57 to PANΔ1 could be due to any one of several non-mutually exclusive models. For example, the ORE may contain a high-affinity binding site for ORF57. Alternatively, there may be a cis-acting sequence in the ORE whose activity is necessary for ORF57 function. As a result, loss of this cis-acting sequence disrupts the ORF57 up-regulatory pathway. Consistent with this idea, previously published data suggested that nt 13–312 contain an activity important for PAN RNA accumulation [53]. Therefore, we examined whether ORF57-responsiveness correlates with the RNA accumulation activity provided by this region of PAN RNA. Using northern blots (data not shown), we confirmed that PANΔ1 is expressed at ∼5-fold lower levels than PAN-WT when ORF57 is not co-expressed (Figure 6B). In contrast, when PANΔ1 is placed behind a CMV_IE_ promoter (CMV-Δ1), the expression levels are reduced only slightly, ∼30% (Figure 6B). Thus, this cis-acting PAN RNA accumulation activity found in nt 13–312 is not operative when a CMV_IE_ promoter is used to express PAN RNA. Using the CMV_IE_-driven PANΔ1 constructs, we next tested the hypothesis that this activity is related to ORF57-responsiveness. Quantitation of northern blot data show that CMV_IE_-driven PANΔ1 remains significantly less responsive to ORF57 than CMV_IE_-driven wild-type PAN RNA (Figure 6C). CMV_IE_-driven WT PAN was up-regulated nearly 10-fold in the presence of ORF57, while the CMV_IE_-driven PANΔ1 RNA levels increase by a factor of only ∼2-fold. Therefore, we conclude that the lack of ORF57-responsiveness of PANΔ1 is unrelated to its reduced levels from the PAN promoter. Moreover, these data show that the ORE functions in a promoter-independent fashion.

### ORF57 binding is sufficient to restore up-regulation to PANΔ1

**Figure 7:**
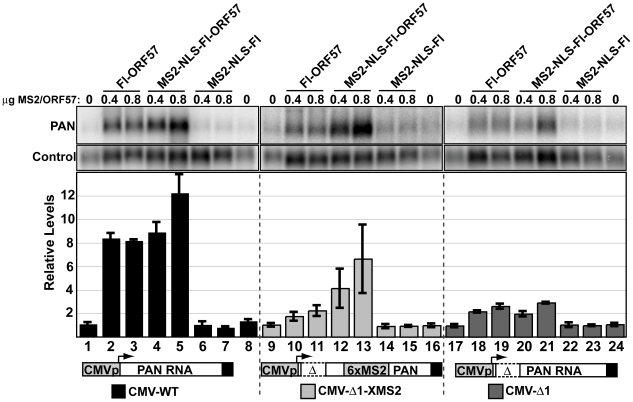
Tethering of ORF57 to CMV-Δ1 restores ORF57-responsiveness. *Top*, northern blots showing levels of the PAN RNA and a control signal from a representative experiment. Fl-ORF57, MS2-NLS-Fl-ORF57, and MS2-NLS-Fl were co-transfected as indicated. The NLS was included to ensure that the MS2 control was appropriately localized to the nucleus. Because the MS2-NLS-Fl-ORF57 expresses at lower levels than Fl-ORF57 (data not shown), the transfection conditions were altered from previous experiments (see Materials and Methods). Average values from three experiments with standard deviations are shown below. The black bars (lanes 1–8), light gray bars (lanes 9–16), and the dark gray bars (lanes 17–24) show relative RNA levels expressed from the CMV-WT, the CMV-Δ1-XMS2, and the CMV-Δ1 plasmid, respectively. These constructs are drawn below (not to scale). In each experiment, the results were normalized to the average value of the “no ORF57” control, which was performed in duplicate (lanes 1,8; 9,16; and 17, 24).

Loss of ORF57 binding to PAN RNA correlates with loss of function, supporting the model that RNA-binding is necessary for ORF57 responsiveness. We next tested whether restoration of ORF57-binding was sufficient to restore ORF57-responsiveness to PANΔ1. To do this, we employed a tethering system using the bacteriophage MS2 coat protein, which binds with high affinity to a well-defined bacteriophage RNA hairpin sequence [59],[60] (Figure 7). We expressed an amino-terminal fusion of the bacteriophage MS2 coat protein with ORF57 (NMS2-NLS-Fl-ORF57) and verified that the fusion did not abrogate ORF57 activity on CMV-driven full-length PAN RNA (lanes 1–8). Expression of this construct increases PAN RNA accumulation similarly to Fl-ORF57, (compare lanes 4 and 5 with 2 and 3), while expression of the MS2-NLS-Fl protein alone did not increase PAN RNA levels (lanes 6 and 7). Therefore, we conclude that NMS2-NLS-Fl-ORF57 maintains ORF57 activity. Next, we co-expressed NMS2-NLS-Fl-ORF57 fusion protein with a CMV-Δ1 derivative that includes six binding sites for the MS2 coat protein (Figure 7, lanes 12,13). In this case, PAN RNA levels increase ∼7-fold. Expression of neither Fl-ORF57 nor MS2-NLS-Fl alone had a similar effect (lanes 10–11 and 14–15, respectively). Importantly, the increase depends on the presence of MS2-binding sites in the RNA: CMV-Δ1 shows minimal response to MS2-NLS-Fl-ORF57 expression (lanes 20, 21). Thus, tethering of ORF57 to PANΔ1 transcripts can complement the lack of ORF57-responsiveness of PANΔ1. We conclude that the stabilization of PAN RNA by ORF57 depends on direct interactions between ORF57 and its target RNA.

### The ORE confers ORF57 responsiveness to an intronless mRNA reporter, but not its spliced counterpart

**Figure 8:**
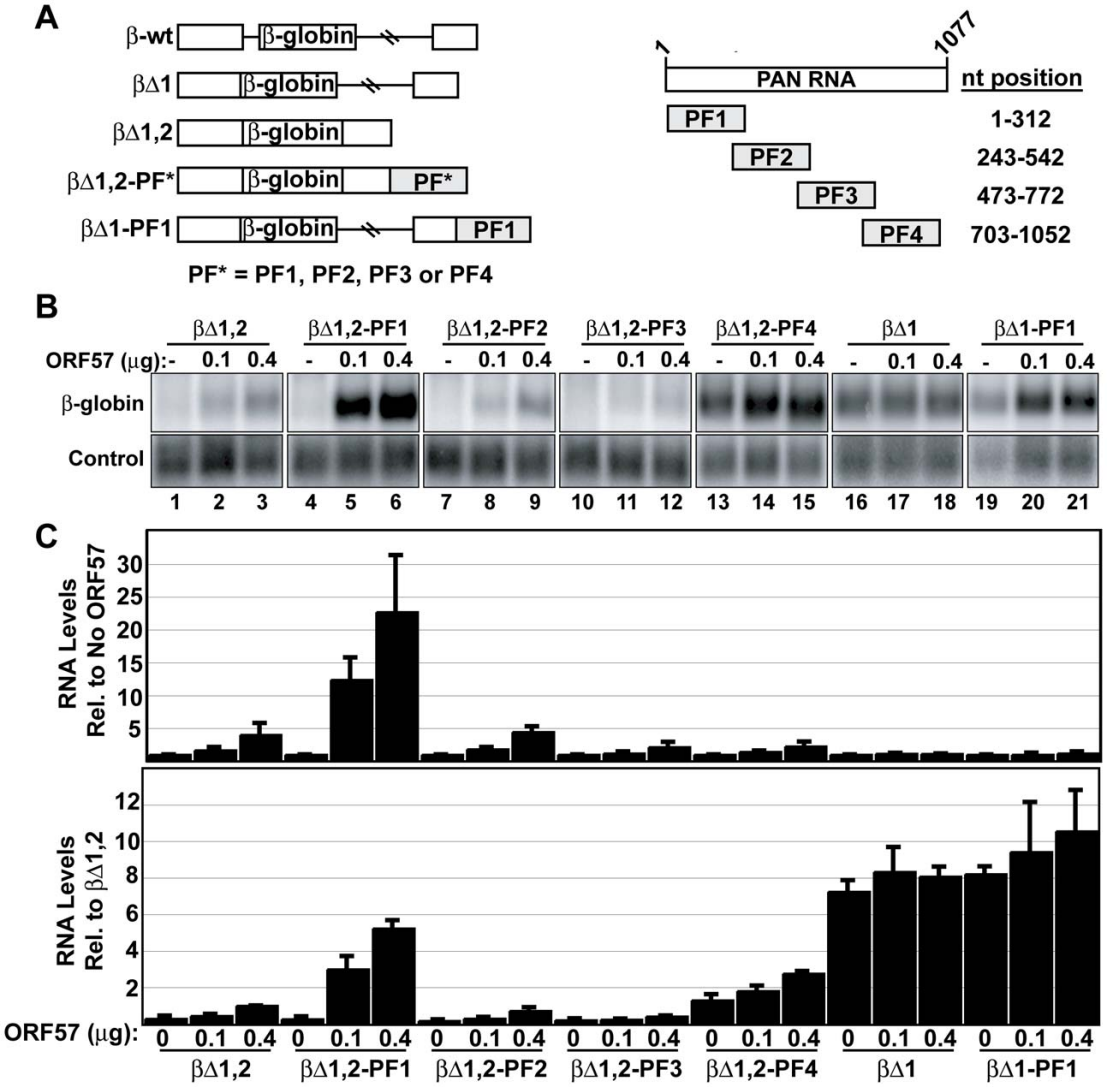
The ORE is sufficient to confer ORF57 responsiveness to an intronless mRNA. (A) *Left*, schematic diagrams of the β-globin reporter constructs described in the text. Each of these constructs is expressed from a CMV_IE_ promoter and has a bovine growth hormone (BGH) polyadenylation signal. The β-globin exonic sequence is shown in white boxes, with horizontal lines representing introns. PF* refers to PF1, PF2, PF3, or PF4. *Right*, schematic representation of the PAN RNA fragments inserted into the β-globin reporters. The nt position is based on the PAN RNA transcription start site. (B) Representative northern blot using total RNA from cells in which β-globin constructs were co-transfected with ORF57 as indicated. These data are all from the same gel and lanes 1–15 are the same exposure; a shorter exposure is shown in lanes 16–21 due to the stronger signal from the spliced mRNAs. The lower panels show a co-transfected control signal. (C) Quantitation of the northern blot data. In the top graph, northern blot data were normalized to the “no ORF57” sample for each set. In the bottom graph, the data were normalized to the βΔ1,2 plus 0.4 µg ORF57 (lane 3) for each experiment. Thus, the top graph shows the ORF57-responsiveness of each transcript, while the bottom graph yields information about the mRNA levels generated from each construct.

The data presented above show that the ORE is necessary for ORF57 responsiveness in the context of PAN RNA. We next tested whether the ORE is sufficient to confer increased ORF57 response to a heterologous transcript. For these experiments, we used a series of previously described β-globin reporter constructs [53]. While wild-type β-globin contains two introns (Figure 8A, β-wt), these reporters delete either the first (βΔ1) or both (βΔ1,2) β-globin introns. Into the 3′ UTR of the βΔ1,2 construct, we cloned ∼300 nt PAN RNA sequences (Figure 8A, right). The sequences, named PF1-PF4 (PAN fragment 1–4), correspond to the sequences deleted in PANΔ1-PANΔ4 (Figure 5C), respectively, except PF1 extends to the 5′-most nucleotide (nt 1-312) and PF4 extends to the nucleotide immediately preceding the polyadenylation hexamer (nt 703–1052). We co-transfected these constructs with the Fl-ORF57 expression plasmid and monitored β-globin mRNA levels by northern blot (Figure 8B, lanes 1–15). Two different schemes were used to normalize the data (Figure 8C). First, to show the response of each mRNA variant to Fl-ORF57, we normalized the β-globin mRNA signal in the presence of Fl-ORF57 to the signal from the same construct in its absence (Figure 8C, top). Second, we normalized all the data to the βΔ1,2 plus 0.4 µg ORF57 (lane 3) to yield information about the expression levels of the reporter mRNAs relative each other (Figure 8C, bottom).

Our data conclusively demonstrate that the ORE increases the response of intronless β-globin mRNA to ORF57. In the absence of any PAN fragment, ORF57 increases the expression levels of intronless β-globin mRNA in a dose-dependent fashion reaching ∼4-fold at the highest ORF57 levels tested (Figure 8B lane 3, Figure 8C). When PF1, which contains the ORE, is placed in the β-globin 3′ UTR, ORF57 has an even greater effect: βΔ1,2-PF1 transcripts are up-regulated ∼23-fold. This effect is specific to PF1 because PF2, PF3, PF4 do not increase ORF57 response. As previously shown [51],[53], the ENE-containing insert (PF4, lanes 13–15) increases the levels of intronless β-globin mRNA. However, as in the case of PAN RNA, ORF57-responsiveness decreases (∼2-fold) when the ENE is included in the transcript (Figure 1). These data show that the ORE is sufficient to confer increased response to ORF57 in a heterologous context. Because the ORE was inserted into the β-globin 3′ UTR, we can further conclude that ORE activity is not strictly dependent upon being at the 5′ end of the transcript.

Published data suggest that intronless mRNAs are subject to degradation by a cellular RNA quality control system [51]–[53],[61],[62] and the data presented above support the model that ORF57 protects transcripts from this RNA decay pathway. If this is the case, we reason that ORF57 should have a limited effect on spliced mRNA abundance, even if the mRNAs contain the ORE, because spliced transcripts are not subject to the cellular RNA quality control pathway. To test this idea, we examined the effects of ORF57 on spliced β-globin mRNA levels containing (βΔ1-PF1) or lacking (βΔ1) the ORE (Figure 8A). Quantitation of β-globin mRNA accumulation by northern blot showed that neither of these mRNAs was significantly altered by ORF57 (Figure 8B, lanes 16–21, and Figure 8C). Examination of the RNA levels shows that the spliced mRNAs accumulate to higher levels than the mRNAs generated from intronless genes, as expected (Figure 8C, bottom). Interestingly, the levels of the ORE-containing intronless transcripts approach those of the spliced mRNAs under the highest levels of ORF57. These results are consistent with the model that ORF57 protects viral mRNAs from cellular RNA decay factors that preferentially degrade transcripts generated from intronless genes.

## Discussion

In this report we uncover three novel findings about the ORF57 protein, an essential KSHV protein involved in viral gene expression. First, these data establish a role for ORF57 in stabilizing nuclear transcripts. Second, our data show that ORF57 binds directly to its target, PAN RNA, in living cells and that binding correlates with function. Third, ORF57 and its homologs can increase the expression of a variety of mRNAs [30]–[33], suggesting a relatively nonspecific effect. However, here we demonstrate the existence of an ORF57-responsive element in PAN RNA suggesting that, at least in some cases, specific cis-acting sequences have evolved to recruit ORF57 to its targets. Consistent with previous reports, our data further show that ORF57 primarily enhances transcript accumulation from intron-lacking genes but not from intron-containing genes, even when the ORE is included in the spliced transcript. Together, these data are consistent with a model in which ORF57 binds to intronless viral transcripts and protects them from a cellular RNA quality control pathway.

The data presented here demonstrate ORF57 stabilizes nuclear RNAs that would otherwise be rapidly degraded. Using steady-state analysis (Figure 1) and a transcription pulse assay (Figure 2), we show that ORF57 increases the stability of an unstable polyadenylated nuclear RNA. Kinetic analysis further supports the conclusion that ORF57 is protecting RNAs, at least in part, from the same rapid RNA decay pathway that the ENE protects transcripts from in cis [51],[52]. Even though ORF57 has been implicated in mRNA export [40]–[42], its expression does not lead to cytoplasmic accumulation of PAN RNA. Thus, we conclude that the effect of ORF57 on RNA stability is independent of its proposed function in RNA export. This conclusion is consistent with previous reports implying that ORF57 stabilizes its target RNAs [39],[42] and with the observations that ORF57 homologs SM and ICP27 can stabilize specific transcripts [63],[64]. However, these data are the first direct demonstration that KSHV ORF57 increases the half-lives of nuclear RNAs and that his function is separable from its proposed roles in mRNA export, transcription, and translation.

We observe only a slight increase in polymerase density on PAN RNA genes driven from either the PAN or tetracycline-responsive promoter (Figure 3). Therefore, we conclude ORF57 increases PAN RNA levels primarily by a post-transcriptional mechanism. We were surprised at the lack of increase in pol II density on the constructs driven by the PAN promoter, which binds the viral transactivator ORF50, an ORF57-interacting protein [38],[39]. Assuming these proteins associate in our experimental system, the interaction appears to have little effect on transcription initiation. It should be noted that we used an antibody (8WG16) that preferentially recognizes the initiating hypophosphorylated form of pol II [65]. Thus, it remains possible that a hyperphosphorylated elongating form of pol II increases on the PAN RNA gene in response to ORF57. Taken with the observation that PAN RNA is up-regulated by ORF57 from four different promoters (Figure 3A), our data strongly support the conclusion that transcription initiation is unaffected by ORF57.

ORF57 enhances the expression of viral mRNAs, the noncoding nuclear PAN RNA, and heterologous reporter mRNAs, so it appeared that its effects were not strongly influenced by cis-acting sequences. On the contrary, our data demonstrate that a specific cis-acting sequence, the ORE, can enhance the effects of ORF57 on both PAN RNA and on a β-globin reporter mRNA. Using a previously described set of deletions [53], we show that ORF57 has reduced binding to PANΔ1 RNA and that this correlates with loss of activity (Figure 5, Figure 6). Moreover, we placed the ORE into an intronless β-globin mRNA and found that it enhances ORF57-responsiveness by ∼5-fold, demonstrating that the ORE affects mRNAs as well as the noncoding PAN RNA (Figure 8). Tethering ORF57 to the ORE-lacking PANΔ1 transcripts in cells is sufficient to complement the ORE deletion, so it seems likely that ORF57 recruitment resides at the core of ORE activity (Figure 7). The simplest interpretation of these results is that the ORE is a high-affinity ORF57-binding site, and that RNA binding by ORF57 is necessary for its stabilization activity. Interestingly, intronless β-globin mRNA levels are enhanced by ORF57 ∼4-fold in the absence of the ORE. Perhaps ORF57 has enough non-specific RNA-binding activity to account for its general effects on reporter RNAs. Alternatively, this effect may be linked to a separate ORF57 activity that functions independently of RNA binding.

Mechanistically, our data support the model that ORF57 binds to its RNA targets and inhibits the activity of nuclear RNA decay enzymes, but we do not yet know the molecular details of ORF57-mediated RNA stabilization. In one model, ORF57 binds RNA making it inaccessible to RNA decay enzymes. Alternatively, ORF57 could indirectly stabilize transcripts by promoting changes in RNP composition or conformation. It remains formally possible that ORF57 increases PAN RNA stability by retaining the transcripts in the nucleus, thereby protecting them from cytoplasmic decay enzymes. However, given the reported role of ORF57 in mRNA export [40]-[42] and its ability to shuttle [66], we think this last hypothesis is unlikely.

Our data are consistent with the model that ORF57 counteracts a nuclear RNA quality control pathway that rapidly degrades transcripts that derived from intronless genes [51]–[53],[61],[62]. It is important to point out that this model does not depend on cells specifically recognizing intronless RNAs. Rather, because of the extensive coupling between the steps of cellular RNA biogenesis including pre-mRNA splicing [16]–[27], we believe it more likely that intronless RNAs are inefficiently processed. If RNA surveillance and RNA maturation are in kinetic competition as formally proposed by Doma and Parker [6], these inefficiently processed intronless transcripts would be predicted to be subject to degradation by RNA quality control pathways. Because the majority of KSHV genes are intronless [14], we propose that ORF57 functions to counteract this RNA decay pathway to promote the robust expression of viral genes. ORF57 has been reported to promote the export of intronless viral mRNAs [40]–[43], so it is easy to imagine that ORF57 allows mRNAs to bypass nuclear decay systems by enhancing the efficiency of their export. However, in the case of PAN RNA, we can uncouple ORF57 mRNA export activities from its function in RNA stabilization. Therefore, our data suggest a more active role for ORF57 in protecting transcripts from degradation in the nucleus. Current work focuses on further testing this model by identifying the cellular decay machinery involved as well as the viral RNAs bound and protected by ORF57.

Because PAN RNA accumulates to such high levels in KSHV lytically reactivated cells [47],[48],[49], it likely performs an important function for the virus. Therefore, studying the biogenesis of this unusual transcript is essential to understanding KSHV biology. In addition, PAN RNA provides a useful a tool to separate ORF57 functions in RNA stability from its role in RNA export. We used an unstable ENE-lacking PAN RNA for our decay studies (Figure 2), but several observations suggest an important role for ORF57 in PAN RNA biogenesis in infected cells. First, steady-state levels of PAN RNA containing the ENE are up-regulated in the presence of ORF57 (Figures 1 and 3). Second, published reports have shown that ORF57-deleted bacmids produce reduced levels of PAN RNA during lytic infection [34],[35]. Finally, we have observed that insertion of multiple copies of the ENE leads to higher levels of PAN RNA (unpublished observations) or βΔ1,2 mRNA [53] than insertion of one ENE, suggesting that a single ENE does not completely block RNA degradation. Thus, the proposed overlapping activities of the ENE and ORF57 may both be essential to fully stabilize PAN RNA during lytic replication. Perhaps more importantly, our studies provide insights into the possible mechanism of ORF57 activity on the accumulation of intronless viral transcripts that lack ENE-like elements. However, further experimentation is necessary to test the role that ORF57 plays on PAN RNA stability in the context of viral infection and on the stability of intronless viral mRNAs.

A particularly interesting component of ORF57-mediated RNA stabilization is the role of the poly(A) tail in regulation of transcript stability. After transcription shut-off in the presence of ORF57, we found that some transcripts become hyperadenylated, while others are partially deadenylated (Figure 2, data not shown). Because both the hypo- and hyperadenylated PAN transcripts are present 8 hrs subsequent to transcription shut-off, it seems that ORF57 stabilizes both forms. Indeed, when we over-expose our northern blots, we observe transcripts resembling these hyper- and hypoadenylated forms in the samples from cells lacking ORF57, but at significantly reduced levels (data not shown). Several different non-exclusive roles for the poly(A) tail in nuclear RNA stability can be imagined that are consistent with our observations. In the first model, PAN RNA is recognized by the cell as an aberrant transcript, presumably due to its lack of export. In manner analogous to yeast and bacterial systems, hyperadenylation of the transcripts is linked to quality control [67],[68]. Interestingly, the host-shutoff mechanism employed by KSHV appears to involve destabilization of hyperadenylated cellular mRNAs [69]. Moreover, recent work implicates mRNA export factors as regulators of poly(A) length in cells and in polyadenylation assays performed in vitro [70]–[73]. In the second model, ORF57 promotes polyadenylation, which then leads to greater transcript stability. In a third model, ORF57 stabilizes nuclear PAN RNA, and the hyperadenylation results from promiscuous polyadenylation of the stabilized nuclear transcripts. Distinguishing among these models will shed light into host-virus interactions between KSHV ORF57, cellular poly(A) machinery, and cellular RNA decay pathways.

## Materials and Methods

### Cell culture, cell lines, and transfection

HEK293, 293TOA, (Clontech) and 293A-TOA cells were grown in Dulbecco's Modified Eagle's Medium (Sigma) supplemented with 10% fetal bovine serum (FBS), 1X penicillin-streptomycin (Sigma), and 2 mM L-glutamate. TOA media utilized tetracycline-free FBS (Clontech) and was supplemented with 100 µg/mL G418 (Fisher Scientific). HH-B2 cells [74] were cultured in RPMI-1640 media (Sigma) supplemented with 15% FBS, 1X penicillin-streptomycin (Sigma), and 2 mM L-glutamate. Transfections were performed using TransIT-293 reagent as per the manufacturer's protocol (Mirus). Most ORF57 titration experiments were performed in 12-well tissue culture plates with a total of 0.7–0.8 µg plasmid DNA. A typical transfection with PAN-promoter constructs contained 0.15 µg of the PAN construct, 0.15 µg of an ORF50 expression construct, which is necessary for transcription from the PAN promoter, 0.1 µg of a control (mgU2-19/30) and 0.4 µg of Fl-ORF57 plus pcDNA3. The control plasmid generates two products; one is a spliced non-coding transcript and the other is an intron-derived scaRNA important for methylation of U2 snRNA [75]. These transcripts show little or no response to ORF57, under our typical transfection conditions. However, they may slightly increase (<2-fold) in the presence of ORF57 under the conditions used in Figure 7 (see below). The result of this slight increase would yield a relative underestimation of the effects of ORF57 in Figure 7, so it does not alter our conclusions. When other promoters were used, 0.3 µg of PAN RNA or β-globin expression plasmids were transfected. Total RNA was harvested 18–24 hrs post-transfection using TRI Reagent (Molecular Research Center). The experiments shown in Figure 7 were slightly different. In this case, we transfected 0.1 µg of the PAN RNA expression plasmids, 0.1 µg of the control plasmid and 0.4 and 0.8 µg of the Fl-ORF57, MS2-NLS-Fl-ORF57, or MS2-NLS-Fl expression constructs. Total RNA was harvested 44–50 hrs post-transfection. The 293A-TOA cell line was generated by transfecting 293A cells (Invitrogen) with a tTA-expressing plasmid (Clontech). Stable transformants were selected in 300 µg/ml G418 and cloned by limiting dilution.

### Decay assays

We have previously described the transcription pulse assay and the quantitation of PAN RNA decay kinetics [51],[52]. We previously used HeLa Tet-off cells, but the expression of ORF57 in HeLa tet-off cells led to abrogation of the tetracycline-responsiveness. The reason for this observation remains unknown. In the current studies, we utilized 293TOA cells.

### Plasmids

PAN-WT, PANΔ1, PANΔ2, PANΔ3, PANΔ4 and β-globin reporters were described in [53], while PAN-Δ79, TRP-WT, and TRP-Δ79 were described in [52]. PcFl-ORF57II (used to express Fl-ORF57) was generated by PCR amplification ORF57 from KSHV DNA using primers NC495 (5′ ATTAGCGGATTCATGGTACAAGCAATGATAGAC 3′) and NC496 (5′ AAAAGGCTCGAGTTAAGAAAGTGGATAAAAGAATAAACCC 3′). Underlined sequence show relevant restriction sites. The product was digested with BamHI and XhoI and inserted into pcDNA-Flag (gift of Jens Lykke-Andersen, University of Colorado) cut with the same. The sequence of all constructs using PCR-based methods was verified. Consistent with previous reports [36],[66], this construct had a silent mutation (CAT to CAC) encoding for the His at amino acid 261.

The CMV_IE_-WT (pcPAN) was made by PCR amplification of PAN transcribed region and downstream sequence using primers NC39 (5′ ATTTCCAAGCTTACTGGGACTGCCCAGTCACCTTGGCTGCCGCTTCACC 3′) and NC42 (5′ TAAAGCGGGCCCCCATCCCAATCGACGCAA 3′). The product was cut with ApaI, blunted, cut with HindIII and inserted into pcDNA3 digested with BbsI, blunted, and then digested with HindIII. The CMV-Δ1 derivative of this construct (pcPANΔ1) was generated by PCR amplification with primers NC494 (5′ CTCCGAAAGCTTACTGGGACTGCCATTCAATC 3′) and NC10 (5′ GGGGGCCCGTCACATTTAGGGCAAAGTGG 3′) using PANΔ1 as a template. The product was digested with XbaI and HindIII and inserted into pcPAN. The SV40 and EF1a-driven constructs were also derivatives of pcPAN. In this case, pcPAN was digested with NruI and HindIII to remove the CMV_IE_ promoter and SV40 or EF1a promoters were inserted using the same restriction sites. Promoter sequences for SV40 and EF1a were amplified using primers NC418 (5′ GATCTCGCGACTCCCCAGGCAGGCAGAAGT 3′) and NC419 (5′ GATCAAGCTTTGGATATACAAGCTCCCGGG 3′), and NC416 (5′ GATCTCGCGAGGCTCCGGTGCCCGTCAGTG 3′) and NC417 (5′ GATCAAGCTTGAACGTTCACGGCGACTACT 3′), respectively.

The NMS2-NLS-Fl expression vector (pcNMS2-NLS-Fl) was constructed by amplifying the MS2 coat protein coding sequence using primers NC448 (5′ AGACCCAAGCTTGCCACCATGGCTTCT 3′) and NC449 (5′ GGATCCAAGCTTAGATCCACCCTTGTCATCGTCGTCCTTGTAGTCTACCTTTCTCTTCTTTTTTGGTCCACCTCCACCTCCGTAGAT 3′) and pcNMS2 [59] as a template. The resulting fragment was digested with HindIII and inserted into pcNMS2 digested with HindIII. NMS2-NLS-Fl-ORF57 was generated by insertion of the BamHI-XhoI fragment of pcFl-ORF57II into pcNMS2-NLS-Fl. The CMV-Δ1-XMS2 construct (pcPANΔ1-XMS2) was generated by amplifying six MS2-binding sites using primers NC584 (5′ AAATGCTCTAGAAACTACCAAACTGGGTCTAG 3′) and NC134 (5′ GACCCTAGATCTACTATAGAATAGGGCCCTCT 3′), digestion of the resulting product with XbaI, and insertion into pcPANΔ1 digested with XbaI.

### ChIP assays

For ChIP assays, one 10 cm plate of HEK293 cells was used (∼10^7^ cells) per sample. Twenty-four hours post-transfection (plus/minus dox as indicated), methanol-free formaldehyde was added to the media at final concentration of 0.75%. Plates were incubated for 10 min and formaldehyde was quenched with 125 mM glycine for 5 min. After washing three times in ice-cold 1X phosphate buffered saline (Sigma), cells were harvested with a rubber policeman and collected by centrifugation at 3500×g for 3 min. Pellets were resuspended in 500 ml RIPA buffer (50 mM Tris-HCl pH 8.0, 150 mM NaCl, 2 mM EDTA, 1% NP-40, 0.5% sodium deoxycholate, 0.1% SDS) with 1X protease inhibitors (cocktail V, Calbiochem), and 1 mM phenylmethanesulphonylfluoride (PMSF), sonicated 6 times for 5 seconds using a Branson Sonifier 450 with a 4.8 mm diameter micro tip producing an average DNA size of ∼250–1000 bp. The extracts were then centrifuged at 800×g for 5 minutes at 4° and the supernatant was pre-cleared for one hour with 20 µl of Protein-A agarose (Pierce). After centrifugation to remove the beads, absorbance 260 was determined. While this does not accurately reflect DNA concentration due to the complexity of the extract, the value can be used to equilibrate extract concentrations for immunoprecipitation (5% was placed at −20° as “input”. Approximately 8 µg of 8WG16 antibody (Abcam) was added to extract (except no antibody control) and the mixture was nutated overnight at 4°. In addition, 20 µl of Protein-A agarose were blocked overnight at 4° with 0.5 mg/ml sheared salmon sperm DNA and 0.1 mg/ml bovine serum albumin (BSA) in RIPA. The next day, the beads were added to the antibody-extract mixture and the nutated for 1.5 hr at 4°. The beads were then washed a total of six times by nutating at room temperature for 3 minutes in 1 mL of the following solutions: 1) RIPA, 2) low salt wash (0.1 SDS, 1% TritonX100, 2 mM EDTA, 20 mM TRIS (pH 8), 150 mM NaCl), 3) high salt wash (0.1 SDS, 1% TritonX100, 2 mM EDTA, 20 mM TRIS (pH 8), 500 mM NaCl), 4) LiCl wash (0.25 M LiCl, 1% NP40, 1% sodium deoxycholate, 1 mM EDTA, 10 mM Tris pH 8.0), 5) TE (10 mM Tris pH 8.0, 1 mM EDTA), 6) TE. After washing, the beads were nutated in 150 mL of elution buffer (1% SDS, 100 mM NaHCO_3_, pH 9.0) for 15 min. The elution step was repeated, the eluted fractions were combined, 60 µl 1M Tris-HCl (pH 6.8) was added to the eluted complexes, proteinase K was added to 0.2 mg/ml and the samples were incubated at 37° for 60 min. The crosslinks were subsequently reversed at 65° for 5–18 hr. The samples were extracted with phenol-chloroform isoamyl alcohol (25∶24∶1), ethanol precipitated in the presence of 0.3M sodium acetate and 20 µg GlycoBlue (Ambion). The pellets washed with 70% ethanol and resuspended in 20 µl of water.

### Real-time PCR and quantitation of ChIP assays

Input and pellet DNA was diluted 1∶100 and 1∶5, respectively and 2 ml of this was used as template for a 20 µl real-time PCR reaction containing iTAQ fast SYBR green supermix (Bio-Rad) with a final concentration of 100 nM primers. Real-time PCR parameters were 40 cycles of 95° for 3 sec and 60° for 30 sec using a 7500 Fast Real-time PCR system (Applied Biosystems). Primers used were: PAN 5′, NC527 (5′ CGCCGATTGTGGGTTGA 3′) and NC528 (5′CGAAAGCCAGGATGGGTATATT 3′); PAN 3′, NC550 (5′ TGTTTTAATGTGTATGTTGTGTTGGAAGT 3′) and NC551 (5′ TTCACCTACAAGAAAACATCGTTAGTC 3′); GAPDH, NC533 (5′ ATGGAAATCCCATCACCATCTT 3′) and NC534 (5′ CTAGTTGCCTCCCCAAAGCA 3′). Efficiency of the PAN 5′, PAN 3′ and GAPDH amplification was determined to be 80%, 63%, and 72%, respectively [76].

Quantitation of the ChIP signals was performed as follows. First, the relative quantities of Inputs and Pellets for each experiment were determined based on their amplification efficiency and the pellet/input ratio was determined. Each experiment included a “no antibody” control and this pellet/input value was subtracted from the other three samples. Subsequently, the background corrected pellet/input PAN 5′ and PAN 3′ ratios were normalized to the corresponding GAPDH ratios for the same experiment and the “no ORF57” values were set to one. The p-values reported throughout the manuscript are unpaired equal variance Student's t-test values.

### RNA immunoprecipitation assays, fluorescence in situ hybridization, northern blotting

UV cross-linking and cell-mixing experiments were performed as previously described [57], except poly(U) was omitted from the protocol. Northern blotting was performed as in [53]. For in situ hybridization analyses, PAN RNA was detected using a mix of three DNA oligonucleotides, NC29 (ATCGGCGGCACCAATGAAAACCAGAAGCGGCAAGAAGGCA), NC30 (CCAATGTTCTTACACGACTTTGAAACTTCTGACAAATGCC), and NC31 (GCACGTTAAATTGTCAAAAGTATAACATGTTTTTCCAATA). In situ hybridization was performed as previously described [53],[77], except in the experiments shown in Figure S3, direct labeling using FAM-conjugated oligonucleotides was employed as an alternative to digoxygenin-tailing protocols. Confocal and fluorescence microscopy was used in Figures 4 and S3, respectively.

## Supporting Information

Table S1 Kinetic constants from PAN RNA decay experiments. These constants were derived from nonlinear regressions of the average values of each data point using the equation y = a*e* ^−bx^+c*e* ^−dx^, where a + c = 100, b<10, b>0, d>0. These data correspond to the decay curves shown in Figure 2B. They differ slightly from the values reported in Figure 2C and Figure S1. These values were derived from a single regression of averaged values for multiple experiments. The data displayed in Figure 2C and Figure S1 were derived from independent regressions of each data set. The latter calculations make it possible to determine standard deviations for each parameter, while the former allow display of a single regression in which each data point is shown with standard deviation.
(0.04 MB DOC)

Figure S1 Kinetic parameters of PAN RNA decay data. (A) Percentage of PAN RNA transcripts in the “slow” decay pathway is shown with standard deviation. These values are effectively the inverse of those displayed in Figure 2C. (B) and (C) are the half-lives determined of the slow and rapid RNA decay pathways, respectively. As previously observed in experiments comparing transcripts containing or lacking the ENE [51], the only parameter consistently significantly affected by ORF57 is the fraction of PAN RNA degrading in each population. Whether the lack of significant differences in half-life determinations between the two pools is due to experimental limitations or reflects a biological phenomenon requires further exploration. Clearly, our data show that the fraction of PAN RNA transcripts that are rapidly degraded decreases when ORF57 is co-expressed.
(0.99 MB TIF)

Figure S2 RNA abundance in the absence of ORF57 from multiple promoters. Quantitation of northern blot data showing PAN RNA levels driven by each of the indicated promoters. All values are relative to the PAN RNA promoter; error bars are standard deviation (*n = 3*). It should be noted that the PAN RNA promoter construct is pBluescript-based while the others are pcDNA3 derivatives, so it is possible that some of the basal accumulation differences are due to this difference. All transcripts utilize the PAN RNA polyadenylation signal.
(0.29 MB TIF)

Figure S3 TRP-Δ79 RNA remains nuclear in the presence of ORF57. TRP-Δ79 was transfected into 293A-TOA cells and the transfected cells were used for in situ hybridization with PAN RNA probes (middle). PAN RNA signal is shown in the presence and absence (vector) of ORF57 as indicated. Nuclei are stained with DAPI (left) and merged images are shown (right panels).
(6.40 MB TIF)
