# Supplementary material for: Kaposi's Sarcoma-Associated Herpesvirus ORF57 Protein Binds and Protects a Nuclear Noncoding RNA from Cellular RNA Decay Pathways
Source: PLoS Pathog. 2010 Mar 5;6(3):e1000799. doi: 10.1371/journal.ppat.1000799 (PMC2832700; doi:10.1371/journal.ppat.1000799)
Supplement: Table S1 — Kinetic constants from PAN RNA decay experiments. These constants were derived from nonlinear regressions of the average values of each data point using the equation y = ae −bx+ce −dx, where a + c = 100, b<10, b>0, d>0. These data correspond to the decay curves shown in Figure 2B. They differ slightly from the values reported in Figure 2C and Figure S1. These values were derived from a single regression of averaged values for multiple experiments. The data displayed in Figure 2C and Figure S1 were derived from independent regressions of each data set. The latter calculations make it possible to determine standard deviations for each parameter, while the former allow display of a single regression in which each data point is shown with standard deviation. (0.04 MB DOC) [file ppat.1000799.s001.doc]

**Table S1.** Kinetic constants from PAN RNA decay experiments.

| **PAN** | **Pulse** | **ORF57** | **a** | **b** | **c** | **d** | **R^2^** | **t_1/2_(fast, min)** | **t_1/2_(slow, hr)** | **% fast** |
| --- | --- | --- | --- | --- | --- | --- | --- | --- | --- | --- |
| Δ79 | 2 | - | 70.1 | 8.4 | 29.1 | 0.220 | 0.997 | 5.0 | 3.2 | 70.1 |
| Δ79 | 2 | + | 50.7 | 4.4 | 49.3 | 0.101 | 0.997 | 9.5 | 6.7 | 50.7 |
| Δ79 | 18 | - | 58.4 | 5.1 | 41.5 | 0.270 | 0.999 | 8.1 | 2.6 | 58.4 |
| Δ79 | 18 | + | 15.4 | 8.8 | 84.6 | 0.213 | 0.982 | 4.7 | 3.3 | 15.4 |

These constants were derived from nonlinear regressions of the average values of each data point using the equation y = a*e*^-bx^ + c*e*^-dx^, where a + c = 100, b < 10, b > 0, d > 0. These data correspond to the decay curves shown in Figure 2B. They differ slightly from the values reported in Figure 2C and Figure S1. These values were derived from a single regression of averaged values for multiple experiments. The data displayed in Figure 2C and Figure S1 were derived from independent regressions of each data set. The latter calculations make it possible to determine standard deviations for each parameter, while the former allow display of a single regression in which each data point is shown with standard deviation.
