# Supplementary figures and images for: Kaposi's Sarcoma-Associated Herpesvirus ORF57 Protein Binds and Protects a Nuclear Noncoding RNA from Cellular RNA Decay Pathways
Source: PLoS Pathog. 2010 Mar 5;6(3):e1000799. doi: 10.1371/journal.ppat.1000799 (PMC2832700; doi:10.1371/journal.ppat.1000799)

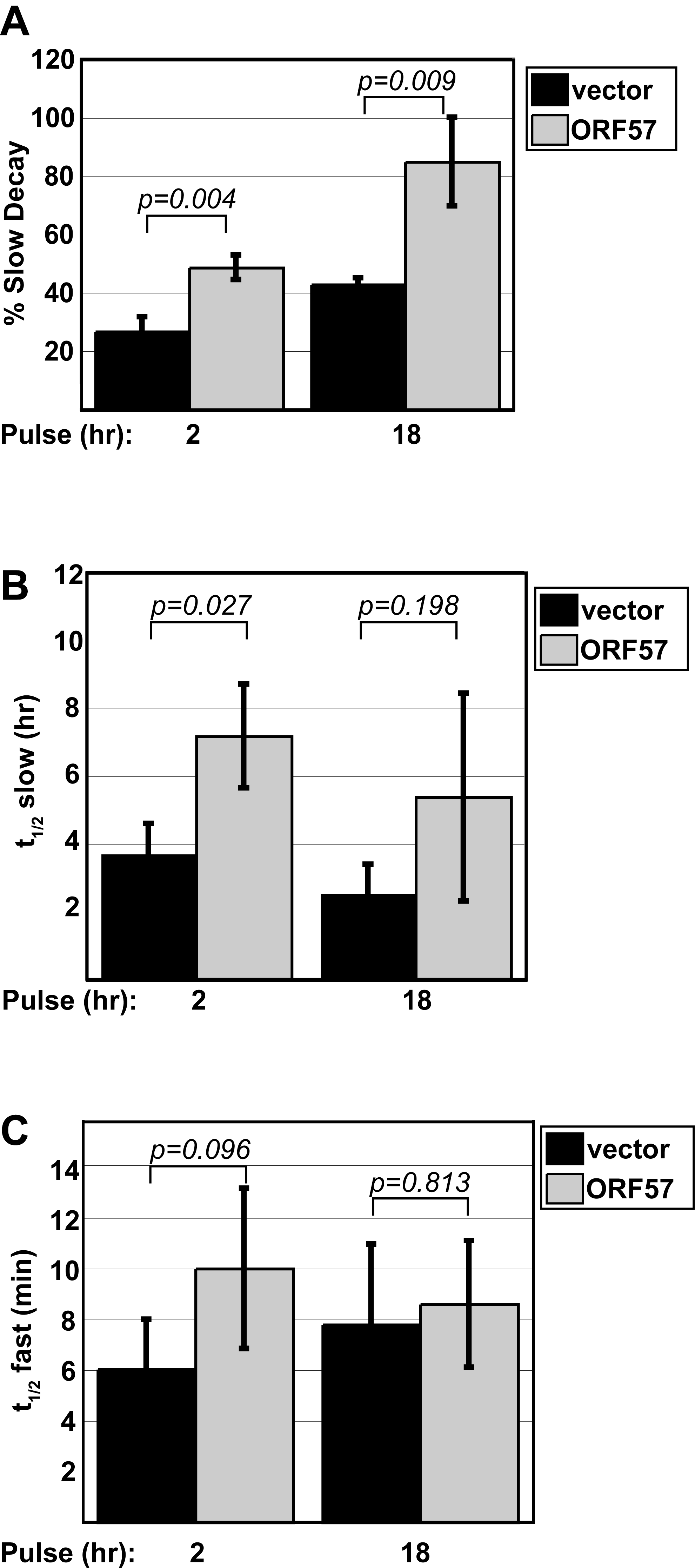

Supplement: Figure S1 — Kinetic parameters of PAN RNA decay data. (A) Percentage of PAN RNA transcripts in the “slow” decay pathway is shown with standard deviation. These values are effectively the inverse of those displayed in Figure 2C. (B) and (C) are the half-lives determined of the slow and rapid RNA decay pathways, respectively. As previously observed in experiments comparing transcripts containing or lacking the ENE [51], the only parameter consistently significantly affected by ORF57 is the fraction of PAN RNA degrading in each population. Whether the lack of significant differences in half-life determinations between the two pools is due to experimental limitations or reflects a biological phenomenon requires further exploration. Clearly, our data show that the fraction of PAN RNA transcripts that are rapidly degraded decreases when ORF57 is co-expressed. (0.99 MB TIF) [file ppat.1000799.s002.tif]

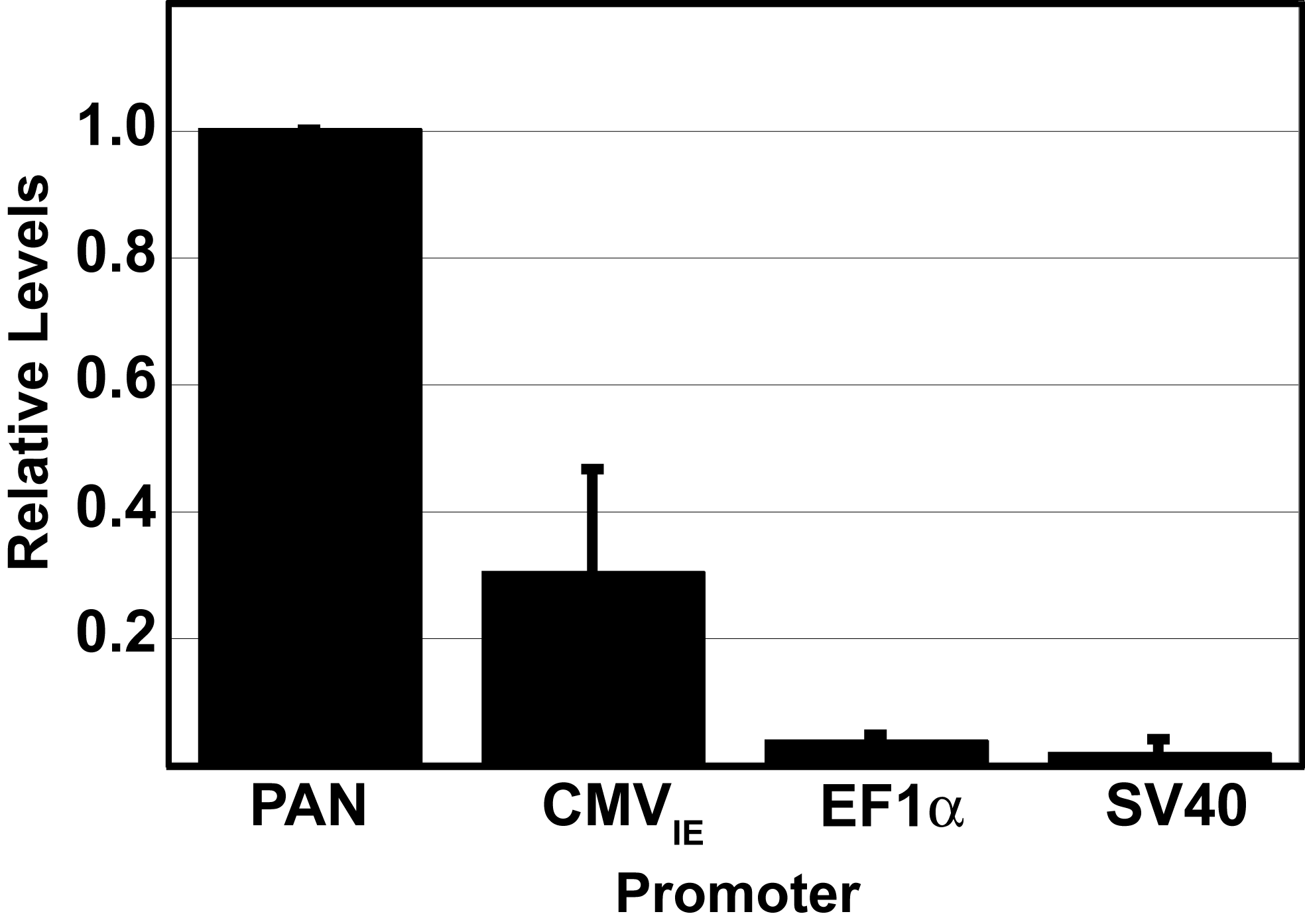

Supplement: Figure S2 — RNA abundance in the absence of ORF57 from multiple promoters. Quantitation of northern blot data showing PAN RNA levels driven by each of the indicated promoters. All values are relative to the PAN RNA promoter; error bars are standard deviation (n = 3). It should be noted that the PAN RNA promoter construct is pBluescript-based while the others are pcDNA3 derivatives, so it is possible that some of the basal accumulation differences are due to this difference. All transcripts utilize the PAN RNA polyadenylation signal. (0.29 MB TIF) [file ppat.1000799.s003.tif]

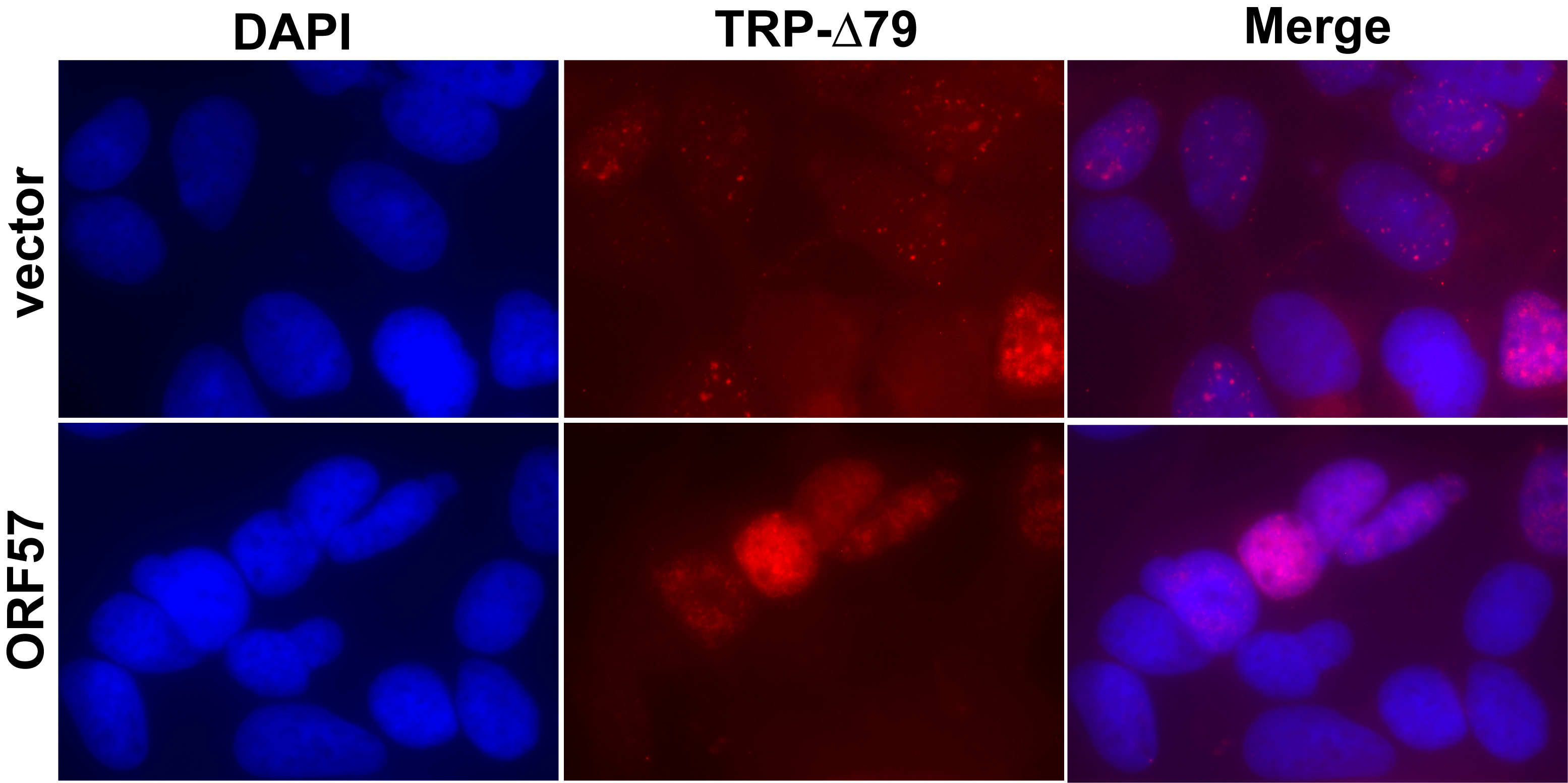

Supplement: Figure S3 — TRP-Δ79 RNA remains nuclear in the presence of ORF57. TRP-Δ79 was transfected into 293A-TOA cells and the transfected cells were used for in situ hybridization with PAN RNA probes (middle). PAN RNA signal is shown in the presence and absence (vector) of ORF57 as indicated. Nuclei are stained with DAPI (left) and merged images are shown (right panels). (6.40 MB TIF) [file ppat.1000799.s004.tif]
